# The impact of school administrators’ influence tactics on teachers’ organizational commitment: the role of learning agility

**DOI:** 10.3389/fpsyg.2025.1549328

**Published:** 2025-04-10

**Authors:** Cemaliye Mahmutoğlu, Cevat Celep, Ayça Kaya

**Affiliations:** ^1^Department of Educational Administration, Supervision and Planning, Faculty of Education, Girne American University, Kyrenia, Cyprus; ^2^Department of Classroom Teaching, Faculty of Education, Girne American University, Kyrenia, Cyprus; ^3^Department of Education Sciences, Haliç University, Istanbul, Türkiye

**Keywords:** influence tactics, organizational commitment, learning agility, school administrators, teachers

## Abstract

This study examines the impact of educational leadership, school administrators’ influence tactics, and teachers’ learning agility on organizational commitment (OC) among primary and secondary school teachers in Northern Cyprus. The research offers significant contributions beyond the Turkish Republic of Northern Cyprus (TRNC), as educational leadership, school administrators’ strategic influence, and learning agility play a crucial role in shaping teachers’ organizational commitment. From an educational management perspective, the study highlights how school leadership practices influence teachers’ OC levels and provides insights that can inform best practices in different educational systems. Educational institutions aiming to enhance teachers’ OC can adapt these findings to develop effective strategies, potentially improving job satisfaction and overall educational quality. Additionally, the study contributes to global educational leadership discussions by offering a comparative perspective on leadership approaches across diverse cultural contexts. The findings from the TRNC case study provide valuable insights into how cultural differences shape leadership strategies and their impact on teaching. As the first study to examine this relationship, it employs multiple regression analysis to investigate the mediating role of learning agility in the effect of influence tactics on OC. The study sample consists of 325 teachers working in primary and secondary schools, with data collected using three validated scales: Teachers’ Influence Tactics, Organizational Commitment (OC), and Learning Agility. The results indicate that school administrators’ influence tactics and teachers’ learning agility significantly impact teachers’ OC. Additionally, school administrators’ direct influence tactics positively affect teachers’ learning agility. Bootstrap technique was used to analyze both direct and indirect effects, revealing that while influence tactics and learning agility positively contribute to OC, the mediating role of learning agility is limited rather than decisive. These findings underscore the strategic importance of leadership behaviors in enhancing teachers’ OC and suggest that learning agility serves as a partial but non-decisive mediator. This study provides an empirical basis for future research examining the interplay between leadership tactics, teacher adaptability, and OC across diverse educational contexts, particularly in post-crisis settings like the pandemic.

## Introduction

School administrators play a pivotal role in shaping the educational environment by guiding teachers toward institutional goals, influencing their behaviors, and fostering organizational commitment. Influence tactics, a critical aspect of leadership, are strategic behaviors administrators employ to persuade and align teachers with institutional objectives (Yukl, 2013). These tactics significantly impact teachers’ organizational commitment, defined as their emotional and professional attachment to the institution (Allen and Meyer, 1990). Learning agility may potentially mediate the relationship between influence tactics and organizational commitment, though its role remains underexplored (DeRue et al., 2012). Research indicates that school principals’ learning agility correlates most strongly with rational decision-making styles, highlighting a preference for analytical approaches in ambiguous situations (Yazıcı, 2024). During crises like the pandemic, school leaders enhanced community resilience through flexibility and forward-thinking, with teachers’ digital competencies and school infrastructure playing critical roles (Raffaghelli and Morselli, 2023). Effective crisis management relies on positive influence tactics, such as rational persuasion and inspirational appeals. Studies show “legitimating” as the most frequently used tactic by principals, with teachers’ commitment peaking in the internalization dimension (Tekben and Koşar, 2019). Soft tactics, like friendliness, enhance teachers’ emotional attachment, while teachers prefer “friendliness” over “coalition” to influence administrators (Yukl, 2013). Influence tactics shape administrators’ ability to guide teachers’ behaviors and emotions, as evidenced by studies on persuasion in organizational settings (Onyekwere, 1989).

This study examines the interplay between school administrators’ influence tactics, teachers’ learning agility, and organizational commitment by grounding its analysis in Yukl’s (2013) Influence Tactics Theory; DeRue et al.’ (2012) Learning Agility Framework; Allen and Meyer’s (1990) Organizational Commitment Model; Deci and Ryan’s (1985) Self-Determination Theory (SDT) and Bandura’s (1977) Social Learning Theory (SLT). Yukl’s (2013) theory systematically categorizes strategic behaviors -such as rational persuasion and inspirational appeals-that leaders employ to shape subordinates’ actions, offering a robust lens to explore how administrators influence teachers’ attitudes and behaviors (Yukl and Falbe, 1990). The Learning Agility Framework, as articulated by DeRue et al. (2012), defines agility as the capacity to learn rapidly and adapt in uncertain contexts, providing a critical construct for understanding teachers’ responsiveness to dynamic educational demands (Mitchinson and Morris, 2014). Allen and Meyer’s (1990) three-component model (affective, continuance, and normative commitment) elucidates how leadership practices shape teachers’ emotional and professional attachment to their institutions (Meyer and Allen, 1997). SDT posits that fulfilling teachers’ basic psychological needs—autonomy, competence, and relatedness—enhances intrinsic motivation, thereby linking administrators’ influence tactics to heightened organizational commitment (Ryan and Deci, 2017). Complementarily, SLT asserts that teachers model their behaviors and attitudes after observing administrators, suggesting that exemplary leadership tactics can foster both learning agility and commitment through social learning processes (Bandura and Walters, 1963). Together, these theories provide a multifaceted conceptual framework that illuminates how administrators’ leadership behaviors influence teachers’ motivational dynamics, adaptive capacities, and commitment levels within educational settings.

Organizational learning mediates the link between organizational intelligence and agility in settings like hospitals, boosting resilience essential for survival (Bahrami et al., 2016). It is defined as a process of sharing knowledge and correcting errors among individuals and teams (Argyris, 1999). Post-pandemic, leaders’ agility offers opportunities to transform school systems by prioritizing strategic goals and systemic integrity (Buffone, 2021). Learning losses during school closures vary with remote education efficacy and parental support, disproportionately affecting disadvantaged students (Dorn et al., 2020). Principals’ learning agility levels are notably high, especially among those with advanced degrees, principals, and increasing experience (Özgenel and Yazıcı, 2021), enabling flexible perspectives in novel situations (DeRue et al., 2012).

In Surakarta, principals’ instructional leadership moderately influences teachers’ commitment (Sukarmin and Sin, 2022), with behaviors beyond time protection and visibility generally viewed positively (Firestone and Pennell, 1993). Teachers’ commitment fluctuates, dipping lowest in late winter, peaking during April testing, and stabilizing through year-end (Price, 2021). Strong, reliable relationships sustain commitment, offering resilience against challenges (Carmeli and Gittell, 2009). Agile leadership traits enhance teachers’ job satisfaction and justice perceptions, with organizational justice mediating this effect (Özgenel et al., 2022). Job satisfaction reflects positive emotional responses to valued job aspects, driving performance (Locke, 1969). Principals frequently use “legitimating,” occasionally employ rational persuasion, encouragement, information, collaboration, appreciation, and consultation, but rarely resort to reciprocity, pressure, personal appeals, or coalitions (Tekben and Koşar, 2019). Highly committed employees work harder and sacrifice more for organizational goals (Allen and Meyer, 1990). Despite the focus on adaptive leadership, the mediating role of learning agility in linking influence tactics to commitment remains underexplored (Hulpia et al., 2012), particularly in educational settings where cultural and contextual factors may shape its effectiveness. This study investigates how administrators’ influence tactics effect teachers’ organizational commitment and whether learning agility mediates this relationship. Building on these theoretical insights, this study seeks to empirically test the direct and indirect effects of influence tactics on organizational commitment, with a specific focus on the potential mediating role of learning agility in a post-pandemic educational context.

## Theoretical framework

### School administrators’ influence tactics

Influence tactics are strategic behaviors that school administrators employ to guide teachers’ actions, shape their attitudes, and ensure alignment with institutional goals (Yukl, 2013). These tactics are generally categorized into hard and soft strategies, each exerting distinct effects in educational settings. Hard tactics, such as coercion and pressure, may lead to resistance and decreased motivation among teachers, whereas soft tactics, including rational persuasion, collaboration, and inspirational appeals, are more effective in fostering teacher engagement and organizational commitment (Dağlı and Çalık, 2016). Although hard tactics can secure short-term compliance, they often diminish morale and create obstacles to maintaining a positive school climate (Moideenkutty and Schmidt, 2011). In contrast, moderate and participatory influence tactics, which provide teachers with greater autonomy and decision-making authority, have been shown to enhance job satisfaction and strengthen organizational commitment (Farmer et al., 1997). To effectively cultivate a supportive and high-performing educational environment, administrators must align their influence tactics with teachers’ values and institutional objectives (Kosar and Pehlivan, 2020). Within this context, school administrators frequently resort to political tactics, such as favoritism, intimidation, and coalition formation, to sustain their influence in educational settings (Tanrıöğen and Kurban, 2017). While certain political strategies can enhance leadership effectiveness, others may generate conflicts and foster resistance among teachers, ultimately weakening organizational commitment.

Furthermore, research suggests that assistant principals, like other mid-level administrators, strategically employ influence tactics to secure support for their initiatives and shape decision-making processes within schools (Maher, 1999). These tactics play a crucial role in helping school administrators navigate institutional hierarchies and ensure alignment with broader educational goals. Among the most frequently employed tactics, consultation—which involves seeking teachers’ input and encouraging participation—has been found to positively correlate with all sub-dimensions of leader-member exchange (LMX). This finding suggests that when school principals actively involve teachers in decision-making, they not only foster stronger professional relationships but also enhance organizational commitment (Özkan and Avan, 2021). Additionally, studies indicate that school principals predominantly utilize rational persuasion, legitimating, and collaboration tactics, while they rarely resort to coercive measures, such as excessive pressure or forming coalitions to enforce compliance (Koçak and Memişoğlu, 2023). Supporting this perspective, a study conducted in secondary schools found that moderate influence tactics contribute to a more efficient work environment and a harmonious school climate while simultaneously enhancing teachers’ organizational citizenship behaviors (Korkmaz and Koşar, 2023). Finally, research on principals leading second-order change in schools suggests that those who successfully implement systemic reforms tend to adopt hybrid influence strategies, combining elements of both soft and hard tactics. In contrast, school leaders who rely solely on soft influence tactics tend to prioritize consultation, inspirational appeals, and rational persuasion to gain teacher support for institutional reforms (Friedman and Berkovich, 2021).

### Teachers’ organizational commitment

Organizational commitment refers to teachers’ level of identification, involvement, and emotional attachment to their institution. It is a crucial factor in ensuring long-term teacher retention, engagement, and performance (Nguni et al., 2006; Katper et al., 2020). Research indicates that school administrators play a vital role in shaping teachers’ organizational commitment through their leadership styles and influence tactics (Hallinger, 2003). In fact, teachers who are committed to their workspace are more inclined to engage in professional development activities that cultivate collaboration (Park et al., 2025). Among various leadership approaches, transformational leadership, participative decision-making, and instructional leadership have been found to be particularly effective in strengthening teacher commitment (Blase and Blase, 2002). School administrators’ leadership behaviors, including shared decision-making and professional support, significantly predict teachers’ organizational commitment (Sokal et al., 2024). These tactics help educators feel more valued and engaged in school development processes, fostering long-term commitment. Furthermore, teachers’ commitment to schools positively relates to both collaboration and teaching practices (Meyer and Allen, 1991). Research further highlights that school administrators who foster trust, encouragement, and a sense of belonging create an environment in which teachers are more likely to remain committed to their institution (Mansor et al., 2021). Distributed leadership and supportive administrative structures positively influence teachers’ engagement and organizational commitment (Hulpia and Devos, 2010).

Moreover, collaboration has been shown to influence various aspects of teacher engagement and well-being. Teacher collaboration significantly enhances teacher efficacy, leading to improvements in instructional practices and student learning outcomes (Park et al., 2025). This suggests that increasing opportunities for collaboration among teachers not only strengthens their commitment but also enhances their instructional clarity and effectiveness. Furthermore, teamwork structures have been shown to play a critical role in teachers’ organizational commitment. The study findings indicate that team teaching had both direct and indirect effects on commitment, with increased teacher empowerment, school communication, and work autonomy contributing to organizational identification (Dee et al., 2006). This supports the idea that collaborative structures in schools can create stronger engagement among educators. Team-based structures, including curriculum development teams and governance teams, provide opportunities for teachers to engage in meaningful decision-making, thereby fostering organizational commitment (Mowday et al., 1982). By integrating effective leadership strategies with a focus on fostering professional development and collaboration, school administrators can enhance teachers’ commitment and ensure a more stable and motivated educational workforce.

### The role of learning agility in the relationship between influence tactics and organizational commitment

Learning agility is a critical factor in today’s dynamic educational landscape, enabling teachers to adapt to new challenges, integrate innovative teaching methods, and respond effectively to evolving institutional demands (Lombardo and Eichinger, 2000). Learning agility consists of four key dimensions that collectively shape teachers’ ability to navigate change: Human relations agility involves actively seeking feedback, considering diverse perspectives, and managing relationships effectively. Teachers with high human relations agility engage positively with students, colleagues, and administrators (Gravett and Caldwell, 2016). Mental agility reflects the ability to analyze complex issues, think critically, and generate innovative solutions, enabling teachers to succeed in rapidly changing educational environments. Change agility represents the capacity to embrace and lead change by experimenting with novel teaching strategies and adjusting to institutional reforms. Teachers with high change agility are more adaptable to evolving educational requirements. Outcome agility refers to the ability to achieve meaningful results in challenging situations, positively influence others, and significantly impact student learning outcomes (DeMeuse et al., 2010). The relationship between school administrators’ influences tactics, teachers’ learning agility, and organizational commitment is crucial in understanding how leadership behaviors can enhance teacher engagement and institutional effectiveness. Studies suggest that learning agility functions as a mediating factor in the link between influence tactics and organizational commitment (Hulpia et al., 2012). In fact, influence tactics play a fundamental role in shaping employees’ perceptions of their organization and can either enhance or diminish their commitment to institutional goals (Vigoda-Gadot and Cohen, 2002). Teachers who exhibit high levels of learning agility are more receptive to participatory and adaptive influence tactics, leading to increased commitment and performance (Hulpia et al., 2012), particularly when administrators foster a collaborative environment that supports professional growth. Moreover, research highlights that the effectiveness of influence tactics is largely dependent on an individual’s ability to align with organizational values and expectations (Ferris and Kacmar, 1992). School administrators who employ expertise-based and participatory tactics can enhance teachers’ adaptability, fostering a culture of continuous learning and commitment (DeMeuse et al., 2010). A recent study also suggests that leaders’ use of influence tactics, particularly apprising and ingratiation, has been shown to impact followers’ affective and normative commitment significantly (Chumbley, 2023). Additionally, transformational leadership positively affects followers’ commitment by instilling a shared vision and engaging them through rational and inspirational influence tactics (Chaturvedi et al., 2019). Despite the growing recognition of learning agility as a key determinant of teacher adaptability and engagement, research examining its role as a mediator between influence tactics and organizational commitment remains limited (Kosar and Pehlivan, 2020), specially in contexts where external disruptions, such as the COVID-19 pandemic, challenge traditional leadership dynamics. Additionally, leadership effectiveness in relation to influence tactics has been a focal point in commitment studies. Transformational leadership retains a relatively high level of importance in fostering affective commitment among employees (Bass, 1990). Furthermore, the effectiveness of influence tactics is dependent on both the leader’s ability to align with organizational values and the employee’s perception of fairness in decision-making (Yukl, 2013). These findings suggest that leadership strategies need to be adaptive to sustain teachers’ organizational commitment and professional growth. Recent findings further highlight that rationality and inspirational appeal partially mediate the relationship between transformational leadership and affective organizational commitment (Chaturvedi et al., 2019). Moreover, research suggests that transformational leadership is distinct from preceding leadership theories because it engages employees through intrinsic motivation and self-concept alignment with organizational goals (Avolio and Bass, 2004). New evidence from the public sector suggests that agile leadership significantly enhances employee commitment by fostering a participatory work culture that emphasizes adaptability and shared decision-making (Lediju, 2016). Additionally, agile leadership fosters learning agility, which in turn acts as a key driver for organizational performance and innovation (Chung, 2024). These insights emphasize that influence tactics must be aligned with teachers’ intrinsic motivation to maximize their organizational commitment. These findings underscore the critical role of leadership approaches not only in enhancing teachers’ organizational commitment but also in fostering institutional adaptability, innovation, and long-term organizational effectiveness.

## The present study

Addressing this gap, the present study examines the extent to which school administrators’ influence tactics impact teachers’ organizational commitment and whether learning agility mediates this relationship. By investigating this connection, the study seeks to provide empirical evidence on the effectiveness of influence tactics in fostering organizational commitment and adaptability among teachers. Based on the theoretical and empirical foundations discussed, the study formulates the following hypotheses:

*H1:* School administrators’ influence tactics directly and positively impact teachers’ organizational commitment.

*H2:* School administrators’ influence tactics indirectly affect teachers’ organizational commitment through learning agility.

*H3:* School administrators’ influence tactics directly and positively impact teachers’ learning agility.

By exploring these interconnections, the study aims to provide practical implications for school administrators, guiding them in developing more effective influence strategies that foster teacher adaptability and long-term institutional commitment.

## Methodology

This study investigates the influence tactics school administrators and teachers use and their effects on organizational commitment. To achieve this, a causal-comparative research design was used to examine cause-and-effect relationships between variables. Specifically, this research used a causal screening model that provided both causal and comparative analysis of the relationship between impact tactics and organizational commitment.

A survey method was used to collect primary data from the target audience. The collected data was then analyzed using the Ordinary Least Squares (OLS) regression method, which is widely used to determine the relationships between dependent and independent variables. In addition to OLS, Bootstrap and Process Macro techniques were used to rigorously test the study’s hypotheses. These methods are particularly effective for testing mediation and moderation models, which are relevant to the current study’s goal of exploring the nuanced effects of influence tactics on organizational justice.

### Study population

The study population of the research consists of teachers working in 1600 primary and secondary schools in the Nicosia, Kyrenia, Güzelyurt, and Lefke regions, according to the statistical yearbook of the Ministry of National Education in the Turkish Republic of Northern Cyprus for the 2021–2022 academic year (Ministry of National Education Statistics Unit, n.d., http://eohd.mebnet.net/?q=IstatistikBiriminu). This includes 355 primary school and 610 secondary school teachers from the Nicosia district, 211 primary school and 140 secondary school teachers from the Girne district, 104 primary school and 115 secondary school teachers from the Guzelyurt district, and 30 primary school and 35 secondary school teachers from the Lefke district. During the period of the research, education was interrupted due to Covid 19 infection. The study population was also taken as a sample because the training was conducted through distance learning and the research data were collected electronically via a Google form. The permission approval document and the announcement text were sent to the schools within the sample by the Ministry of National Education and Culture, and an announcement text, including the research link, was sent to the researcher through the WhatsApp communication groups of the schools. Within the sample, 387 teachers completed the survey. Due to some incomplete and incorrect procedures during the preliminary examination, 325 questionnaires were evaluated. To collect data in the study, three different measurement tools were used to determine influence tactics, learning agility and teachers’ organizational commitment.

### Profile of participants

In Table 1, the demographic characteristics of the participants (gender and age distribution) and the distribution profile according to their occupational characteristics in education (school type, professional experience, and school experience) are presented.

**Table 1:** Demographic and professional characteristics of the participants in the academic arena.

Variable	Type	Frequency	Percent
Gender	Female	218	67.1
	Male	107	32.9
Age	20–30	45	13.8
	31–40	111	34.2
	41–50	117	36.0
	61 and above	51	15.7
School Type	Primary School	131	40.3
	Middle School	45	13.8
	General High School	114	35.1
	Vocational High School	35	10.8
Professional Experience	Less than 1 year	11	3.4
	1–5 years	38	11.7
	6–10 years	49	15.1
	11–15 years	53	16.3
	16–20 years	56	17.2
	21–25 years	68	20.9
	26 years and more	50	15.4
School Experience	1–5 years	69	21,2
	6–10 years	109	33,5
	11–15 years	33	10,2
	16–20 years	38	11,7
	21–25 years	33	10,2
	26 years and more	27	8,3
	1–5 years	16	4,9

This stratification emphasizes the balanced representation of participants from both the primary and secondary levels, allowing for a comprehensive analysis of influence tactics and their impact on organizational commitment in different school settings.

### Data collection tools

This study used three different scales to evaluate the influence of tactics of school administrators, learning agility, and teachers’ organizational commitment. These tools provided extensive data for the analysis of the study:

#### Administrators’ teacher influence tactics scale

“Administrators’ Teacher Influence Tactics Scale” was developed by the researcher and aims to examine the methods of influencing teachers. This scale consists of 7 dimensions and 34 questions in total. In addition, it was combined with the “Employee Affected Behavior Scale,” which consists of 11 dimensions and 44 questions, adapted from Gary Yulk by Büyükgöze and Özdemir (2019). This combination provides comprehensive data to better understand the impact administrators have on teachers. This scale, developed by Celep and Kaya (2024), measures the influence tactics used by school administrators. It comprises 46 items across six dimensions: Rule, Authority, Expertise, Mutual Benefit, Reward, and Relationship. These dimensions identify various strategies administrators use to influence teachers, reflecting the different forms of power and interpersonal tactics used in the school environment.

#### Teachers’ organizational commitment scale

Two scales were used to measure organizational commitment based on whether commitment was focused on in-school or out-of-school structures. The Focus Scale of In-School Dedication developed by Celep (1996) consists of 29 items distributed in two dimensions: dedication to the school and the teaching profession. This scale examines teachers’ commitment to their schools and teaching roles. The Teachers’ Focus on Out-of-School Dedication Scale (Celep and Bulbul, 2003) for out-of-school dedication consisted of 10 items under a single dimension. This scale shows how much teachers prioritize commitments outside of the school context.

#### Learning agility scale

The scale consists of 1 dimension and 10 items. The scale developed by Gravett and Caldwell (2016) was adapted to Türkiye by Kaya and Argon (2023). The scale displays a 4-factor structure consisting of 23 items. These factors are called ‘mental agility’ (6 items), ‘Human Relations Agility’ (6 items), ‘Change Agility’ (6 items), ‘agility to focus on results’ (5 items). Items 4, 6 and 14 were reverse scored. Examples of statements in the scale include *‘I am optimistic that I can learn new information’; “I enjoy researching new information.”* The scale is a 5-point Likert-type scale ranging from 1 (rarely) to 5 (always). While a score ranking between 6 and 30 was made for the dimensions of mental agility, human relations agility and change agility, a score ranking between 7 and 35 was made in the agility dimension to focus on the results. The higher the score, the higher the agility rating. The scale has three score ranges. 18 and under is referred to as low-level agility, 19–24 as intermediate agility, and 25 or more as high-level agility. Cronbach’s internal consistency coefficient of the scale α = 0.92.

## Results

A correlation analysis was conducted to examine the relationship between school administrators’ influence tactics and teachers’ organizational commitment through the mediating role of learning agility. Specifically, due to the abnormal distribution of the data, non-parametric Spearman correlation analysis was used. This statistical method is useful for assessing the strength and direction of the relationship between variables, even if the data does not meet normality assumptions.

### Descriptive statistics

Table 2 presents descriptive statistics for the variables. These statistics provide an overview of the central trend (mean) and variability (standard deviation) of key variables related to influence tactics, learning agility, and organizational commitment.

**Table 2:** Descriptive statistics on variables.

Variable	*N*	Mean (x̄)	SD	Min	Max
Official Rules	325	3.401	0.784	1.00	5.00
The Power of Authority	325	1.803	0.770	1.00	4.80
Expertise	325	3.501	0.763	1.00	5.00
Mutual Benefit	325	2.296	0.914	1.00	5.00
Prize	325	2.531	0.783	1.00	5.00
Relation	325	2.788	0.779	1.00	5.00
Mental Agility	325	4.092	0.537	2.33	5.00
Human Relationship Agility	325	3.988	0.592	1.83	5.00
Change Agility	325	3.339	0.522	2.00	5.00
Result-Oriented Agility	325	3.865	0.497	1.40	5.00
Commitment to School	325	3.936	0.623	2.20	5.00
Commitment to Politics	325	2.338	0.705	1.00	4.60
Commitment to the Teaching Profession	325	4.303	0.449	2.50	5.00

The results show that among the sub-dimensions of organizational commitment, the highest average score was for commitment to the teaching profession (x̄=4.303), indicating a strong professional identity among teachers. In contrast, the lowest average score was for authority power (x̄=1.803), suggesting that teachers responded less favorably to hierarchical tactics, possibly due to altered dynamics during the pandemic’s shift to distance learning.

Other notable findings include high average scores on school dedication, mental agility, human relations agility, results-oriented agility, rule-oriented, and expertise-oriented tactics. These results suggest that teachers demonstrate a solid commitment to their school and profession and a high level of adherence and engagement to rules and expertise-based influence tactics.

Lower average scores were observed for change agility and relationship-oriented tactics, suggesting that these dimensions may need further development. The results also point to a moderate political commitment and relationship-driven correlation, highlighting the complex interplay between personal, professional, and organizational commitments among teachers.

### Correlation analysis

In this study, Spearman’s correlation analysis examined the relationships between organizational commitment, learning agility, and influence tactics used by school administrators. The results of this analysis are presented in Table 3, which summarizes the correlations between the sub-dimensions of organizational commitment, learning agility, and influence tactics.

**Table 3:** Spearman correlation analysis of organizational commitment, learning agility and influence tactics.

Variable	1	2	3	4	5	6	7	8	9	10	11	12	13
Commitment to School	1.000												
Commitment to Politics	−0.243**	1.000											
Commitment to the Teaching Profession	0.394**	−0.079	1.000										
Mental Agility	0.108	−0.179**	0.166**	1.000									
Human Relationship Agility	0.059	−0.076	0.216**	0.330**	1.000								
Change Agility	0.144**	−0.058	0.034	0.041	0.414**	1.000							
Result-Oriented Agility	0.065	−0.115*	0.134*	0.278**	0.425**	0.486**	1.000						
Official Rules	0.118*	−0.085	0.184**	0.168**	0.140*	0.053	0.060	1.000					
The Power of Authority	−0.299**	0.179**	−0.127*	−0.144**	−0.011	−0.021	−0.030	0.153**	1.000				
Expertise	0.275**	−0.031	0.237**	0.226**	0.232**	0.032	0.066	0.382**	−0.117*	1.000			
Mutual Benefit	−0.016	0.205**	0.020	−0.212**	0.057	0.123*	0.024	0.074	0.268**	0.106	1.000		
Prize	0.136*	0.192**	0.134*	−0.104	0.102	0.149**	0.104	0.058	0.035	0.331**	0.535**	1.000	
Relation	0.106	0.081	0.030	−0.141*	0.135*	0.131*	0.024	0.134*	−0.057	0.282**	0.449**	0.613**	1.000

**N = 325. *p < 0.05; **p < 0.01.

The results in Table 3 reveal several significant correlations between the study variables:

- Organizational Commitment and Learning Agility: There is a significant positive relationship between commitment to the teaching profession and both mental agility (r = 0.166, *p* < 0.01) and human relations agility (r = 0.216, *p* < 0.01). This suggests that teachers with higher learning agility, especially cognitive and interpersonal flexibility, tend to be more committed to their teaching profession.
- Influence Tactics and Organizational Commitment: Expertise-based influence tactics were positively correlated with commitment to the teaching profession (r = 0.237, *p* < 0.01) and school commitment (r = 0.275, *p* < 0.01). On the other hand, authority power shows a significant negative correlation with school commitment (r = −0.299, *p* < 0.01), indicating that relying on formal authority may reduce teachers’ commitment to their schools.
- Learning Agility and Influence Tactics: Sub-dimensions of learning agility, such as human relationship agility, mental agility, and result-oriented agility, are positively correlated with expertise-based influence tactics (r = 0.226, *p* < 0.01), indicating that school administrators who demonstrate expertise are more likely to improve learning agility among their teachers.

These findings support the hypothesis that learning agility significantly mediates the relationship between impact tactics and organizational commitment.

### Regression analysis

A regression analysis using the bootstrap method was conducted to examine whether learning agility mediates the relationship between school administrators’ influence tactics and teachers’ organizational commitment. This analysis aimed to test the direct and indirect effects of influence tactics on organizational commitment through the mediating role of learning agility. The study investigated the impact of influence tactics on organizational commitment, with learning agility as the mediator variable.

**Figure 1:**
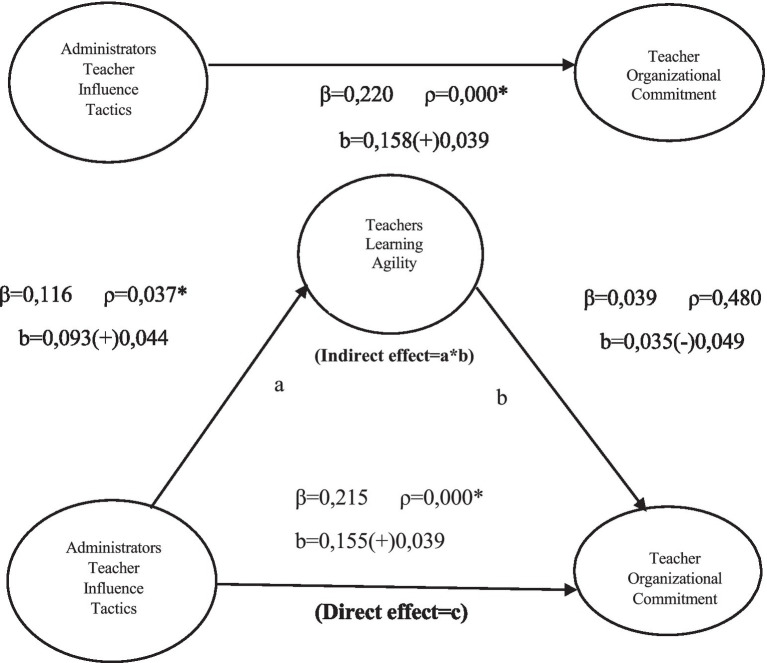
The effect model of managers influence teacher’s behavior on organizational commitment via teachers’ learning agility tool variable.

In Figure 1, the regression analysis using the bootstrap method illustrates the direct and indirect effects of “school administrators’ influence tactics on teachers” on “organizational commitment” via the mediating variable of “learning agility.”

The analysis used Hayes’ (2018) Process Macro, with 5,000 bootstrap resamples selected. The 95% confidence interval (CI) should not include zero for the mediation effect to be considered significant. This study also emphasizes the importance of examining the direct and indirect impact on the dependent variable to highlight the mediating role. The indirect effect of influence tactics on organizational commitment through learning agility was reviewed to assess further the potential mediating role of learning agility. The statistical significance of the mediation effect was determined by analyzing BootLLCI (Lower-Level Confidence Interval) and BootULCI (Upper-Level Confidence Interval) values using Hayes’ (2018) SPSS Process Macro. For the mediation effect to be considered significant, both the BootLLCI and BootULCI values must be in the same direction (either positive or negative). The mediation effect is not statistically significant if the confidence intervals include zero.

The findings indicate that learning agility does not fully mediate the relationship between influence tactics and organizational commitment. While the direct effect of influence tactics on organizational commitment was significant (*p* < 0.05), learning agility did not act as a full mediator in this relationship. The bootstrap analysis results show that learning agility does not meet the criteria to function as a significant mediator in this relationship (Effect = 0.003; BootLLCI = −0.005; BootULCI = 0.015). Since the confidence intervals include zero, learning agility’s mediating effect between influence tactics and organizational commitment is not statistically significant.

Table 4 shows that the indirect effect of influence tactics on organizational commitment through learning agility is not statistically significant.

**Table 4:** The indirect effect of influence tactics on organizational commitment through learning agility.

Indirect effect (a*b)	Effect	S.E. Mean	Lower CI	Upper CI
Influence Tactics → Learning Agility → Organizational Commitment	0.003	0.005	−0.005	0.015

As shown in Table 5, the direct effect of influence tactics on organizational commitment is statistically significant (*p* < 0.001). The confidence interval (CI: 0.077, 0.232) supports this result, demonstrating a significant direct effect of influence tactics on organizational commitment.

**Table 5:** The direct effect of influence tactics on organizational commitment.

Direct effect (c)	Effect	S.E. Mean	*t*	*p*	Lower CI	Upper CI
Influence Tactics → Organizational Commitment	0.154	0.039	3.938	0.000	0.077	0.232

In conclusion, although learning agility has some effect on the relationship between influence tactics and organizational commitment, it cannot be considered a full mediator. Further research is recommended to explore alternative mediators or moderators that may better explain the relationship between these variables, enhancing our understanding of these dynamics.

### Hypothesis testing

Table 6 presents the hypothesis test results in our study.

**Table 6:** Hypothesis test results.

Hypothesis	Beta	*p*-value	Significant (*p* < 0.05)
**H01**: Influence tactics directly and positively impact teachers’ organizational commitment.	0.215	0.000	Yes
**H02**: Influence tactics indirectly negatively affect teachers’ organizational commitment through learning agility.	0.039	0.480	No
**H03**: Influence tactics directly and positively impact teachers’ learning agility.	0.116	0.037	Yes

H01 was accepted (*p* < 0.001), which confirmed that influence tactics directly and positively impact teachers’ organizational commitment. This finding highlights the importance of school administrators’ influence tactics in fostering engagement among teachers.

H02 was rejected (*p* > 0.05), indicating that learning agility does not mediate the relationship between impact tactics and organizational commitment. This suggests that while influence tactics directly affect organizational commitment, learning agility does not mediate.

Acceptance of H03 (*p* < 0.05) supports the hypothesis that influence tactics directly and positively affect teachers’ learning agility. This finding highlights that administrators’ effective use of influence tactics can improve teachers’ ability to learn and adapt in dynamic environments.

The results of this study confirm that the influence tactics used by school administrators have a significant direct impact on teachers’ organizational commitment. Although learning agility has been found to have a positive relationship with both influence tactics and organizational commitment, it does not serve as a significant mediating variable in this relationship. However, the direct positive impact of influence tactics on learning agility suggests that school administrators can improve teachers’ adaptability and performance by strategically using tactics. Further research may explore additional mediators or regulatory factors that may enhance our understanding of these relationships in educational settings.

## Discussion

This study aimed to investigate the tactics used by school administrators and their impact on teachers’ organizational commitment, focusing on the mediating role of learning agility. Using a causal-comparative design, the research surveyed teachers working in primary and secondary schools in Northern Cyprus. The findings highlight the crucial role of school administrators’ influence tactics in shaping teachers’ organizational commitment and learning agility. Leadership style is primarily determined by the sources of power used to guide employees toward organizational goals. The study confirms that influence tactics are central to effective leadership (McShane and Von Glinow, 2017) and that both strict and moderate tactics impact employee motivation and behavior (Dağlı and Çalık, 2016). These results align with leadership theories emphasizing support and supervision as key leadership functions (Bass, 1985; Burns, 1978; Hallinger and Murphy, 1985).

Descriptive analysis findings indicate that teachers show the highest commitment to the teaching profession, supporting the idea that professional identity serves as a strong motivational factor (Allen and Meyer, 1990). In contrast, authority-based influence tactics received the lowest mean scores, suggesting that teachers respond more favorably to leadership approaches based on expertise and knowledge rather than formal authority (Podsakoff et al., 1996). Additionally, the high scores of expertise and rule-based influence tactics indicate that teachers value structured guidance and professional competence (Yukl, 2013).

Correlation analyses reveal that school administrators’ expertise-based influence tactics are positively associated with teachers’ organizational commitment (Schriesheim et al., 1999). Conversely, authority-based influence tactics demonstrate a negative correlation with organizational commitment. This finding aligns with previous research, which indicates that coercive and hierarchical leadership strategies may reduce employees’ commitment (Bass and Riggio, 2006). Furthermore, the positive relationship between learning agility and expertise-based influence tactics suggests that leaders’ knowledge and skills contribute to enhancing teachers’ adaptability (DeMeuse et al., 2010).

Regression analysis results indicate that influence tactics have a direct and significant impact on teachers’ organizational commitment (Geijsel et al., 2003). However, learning agility did not fully mediate this relationship. This finding implies that while learning agility enhances adaptability, organizational commitment in educational settings may be more strongly influenced by contextual factors such as school culture, leadership trust, and teacher autonomy (Judge and Piccolo, 2004). Specifically, the findings confirm that when school administrators use expertise-based influence tactics, teachers’ organizational commitment increases, whereas authority-based leadership strategies negatively affect commitment (Freeman and Fields, 2020).

Hypothesis 1 was confirmed, demonstrating that school administrators’ influence tactics significantly impact teachers’ organizational commitment. The direct relationship between influence tactics, particularly expertise-based approaches, and organizational commitment is a key finding of this study. Expertise as an influence tactic positively correlates with commitment to both the school and the teaching profession, consistent with past research (Podsakoff et al., 1996; Yukl, 2013). Leaders who use specialization and professional expertise influence employees through knowledge rather than authority or coercion, which strengthens organizational commitment. Prior studies also emphasize that influence tactics based on knowledge enhance confidence in leadership and organizational identification (Schriesheim et al., 1999), both of which are essential for long-term commitment. Findings further indicate that transactional leadership styles based on hierarchical authority negatively impact intrinsic motivation and commitment (Bass and Riggio, 2006). Additionally, transformational leadership, which focuses on motivating and inspiring staff, is linked to increased organizational commitment (Magallanes and Dioso, 2020; Prempeh and Kim, 2022). Administrators using participatory and flexible influence tactics, promoting collaboration and teacher autonomy, foster stronger organizational commitment (Atalay and Kanmaz, 2023). Conversely, authoritarian or coercive tactics can demoralize teachers and reduce engagement (Freeman and Fields, 2020).

Hypothesis 2 was only partially supported, indicating that learning agility does not fully mediate the relationship between influence tactics and organizational commitment. While learning agility was positively associated with influence tactics and organizational commitment, it was not a significant mediator. This challenges previous research emphasizing adaptability as a key factor in leadership effectiveness (DeRue et al., 2012; Dries, 2013). Learning agility allows teachers to navigate challenges such as curriculum changes and shifting administrative demands (Kaiser and Overfield, 2010; Swisher and Dai, 2014). However, in educational settings, school culture, leadership trust, and goal alignment may play more critical roles in mediating the relationship between influence tactics and commitment (Judge and Piccolo, 2004; Thien and Liu, 2024). Prior studies indicate that teachers with higher learning agility are more engaged in leadership initiatives and organizational commitment (Paletta et al., 2020), yet this study finds that while learning agility supports adaptability, it does not fully mediate the effect of influence tactics on engagement.

Hypothesis 3 was also confirmed, demonstrating that organizational commitment is shaped not only by influence tactics but also by external factors such as school culture and teacher autonomy. Studies emphasize the role of professional development opportunities and collaborative environments in fostering commitment (Atalay and Kanmaz, 2023). Schools where administrators cultivate a participatory culture exhibit higher levels of teacher commitment. Prior research suggests that more experienced teachers or those in smaller schools respond more positively to participatory leadership approaches (Kosar and Pehlivan, 2020), highlighting the importance of context-specific leadership strategies. The study also underscores the multidimensional nature of organizational commitment, encompassing commitment to the organization, the profession, and broader institutional factors (Meyer and Allen, 1991). Teachers often identify more strongly with their professional roles than their schools, driven by intrinsic motivation and dedication to student achievement (Day et al., 2005; Firestone and Pennell, 1993). However, commitment to a specific school can be influenced by leadership style, organizational justice, and workplace relationships (Lipsky, 2010). The study confirms that school administrators’ leadership behaviors significantly impact teachers’ school commitment, consistent with findings from prior research on the importance of leadership in shaping teacher engagement (Fullan, 2001).

Furthermore, this study finds that expertise-based influence tactics are more effective in increasing organizational commitment than hierarchical authority or transactional leadership. Transformational leadership, which inspires and motivates teachers, fosters greater commitment (Geijsel et al., 2003). Leadership strategies that emphasize collaboration, persuasion, and expertise positively impact organizational commitment (Magallanes and Dioso, 2020). Additionally, professional development programs that promote learning agility can support long-term commitment (Tschannen-Moran and Barr, 2004). However, coercive leadership styles are linked to lower job satisfaction and higher teacher turnover rates (Ingersoll, 2001), reinforcing the need for school administrators to focus on knowledge-based influence and participatory decision-making.

## Conclusion

This study underscores the critical role of leadership influence tactics in shaping teachers’ organizational commitment. The findings reveal that expertise-based influence tactics positively enhance commitment, while authority-based approaches have a negative impact. Although learning agility was positively associated with both influence tactics and organizational commitment, it did not mediate the relationship as initially hypothesized. This suggests that other contextual factors, such as school culture, teacher autonomy, and organizational justice, may play a more significant role in this dynamic. Additionally, the findings indicate that the effectiveness of leadership influence tactics may vary depending on teachers’ experience levels and school environments, underscoring the importance of tailoring leadership strategies to specific institutional and cultural contexts. The results highlight the need for adaptable leadership strategies that foster professional development, trust, and collaboration within schools. School administrators who emphasize expertise, confidence, and participatory decision-making can create a more engaging and resilient educational environment. Encouraging teachers’ professional growth through supportive leadership is essential for sustaining high levels of organizational commitment and improving overall school performance. Furthermore, leadership approaches that promote collaboration and shared decision-making have been found to contribute to long-term teacher retention and job satisfaction, reinforcing the importance of inclusive leadership models.

Future research should further investigate alternative mediators and moderators that may better explain the relationship between influence tactics and organizational commitment. Longitudinal studies could provide deeper insights into how these dynamics evolve over time, particularly in response to educational reforms or leadership transitions. Additionally, exploring the role of external factors, such as policy changes and institutional support systems, could contribute to a more comprehensive understanding of leadership effectiveness in educational settings. By advancing knowledge on the interplay between leadership styles, learning agility, and organizational commitment, this study contributes to ongoing discussions in educational leadership. A continued focus on evidence-based leadership practices will be essential for fostering teacher engagement, enhancing institutional stability, and promoting sustainable improvements in education systems.

### Limitations

This study is subject to several limitations that should be acknowledged. First, the sample was confined to 325 teachers from primary and secondary schools in Northern Cyprus, representing a relatively small and geographically specific population, which restricts the generalizability of the findings to other educational contexts or cultural settings. Second, data collection occurred electronically via Google Forms during the COVID-19 pandemic, a period when education shifted to distance learning, potentially altering administrator-teacher interactions and teachers’ perceptions of influence tactics and organizational commitment. Third, the cross-sectional design limits the ability to assess the long-term effects of influence tactics and learning agility on organizational commitment, particularly under varying conditions such as post-pandemic recovery. Fourth, the finding that learning agility did not significantly mediate the relationship between influence tactics and organizational commitment suggests potential constraints in the conceptualization or measurement of learning agility within this study, possibly due to the adapted scale’s applicability to the educational context or the specific tactics assessed. Finally, the reliance on self-reported survey data introduces the risk of response bias, as teachers’ perceptions may not fully reflect actual leadership behaviors or their objective impact on commitment.

### Suggestions for future research

Future research should address these limitations by expanding the sample size and including diverse educational contexts, such as urban and rural schools or public and private institutions across different countries, to enhance the generalizability of the findings and further explore the mediating role of learning agility. Given the study’s context during the COVID-19 pandemic, investigations comparing the effects of influence tactics in traditional face-to-face settings versus remote environments could clarify whether in-person interactions strengthen organizational commitment more effectively than digital communication. Since learning agility did not emerge as a significant mediator, future studies should examine alternative mediators—such as trust in leadership, perceived organizational justice, or teacher autonomy—to provide a more nuanced understanding of how influence tactics shape organizational commitment. For instance, trust could be tested as a mediator between expertise-based tactics and affective commitment, building on the study’s correlation findings. Additionally, longitudinal designs are recommended to track the sustained impact of influence tactics and learning agility on organizational commitment over time, particularly in response to educational disruptions or reforms. Exploring the role of specific leadership styles, such as transformational or distributed leadership, could also deepen insights into how administrators’ behaviors enhance teachers’ adaptability and engagement, especially given the positive direct effects of expertise-based tactics observed in this study. Finally, incorporating mixed-method approaches, combining quantitative surveys with qualitative interviews, could offer a richer perspective on how teachers perceive and respond to influence tactics, addressing potential biases in self-reported data and enriching the understanding of leadership dynamics in education.

## Data Availability

The original contributions presented in the study are included in the article/supplementary material, further inquiries can be directed to the corresponding author.
